# Metabolic Responses of Poplar to *Apripona germari* (Hope) as Revealed by Metabolite Profiling

**DOI:** 10.3390/ijms17060923

**Published:** 2016-06-20

**Authors:** Lijuan Wang, Liangjian Qu, Liwei Zhang, Jianjun Hu, Fang Tang, Mengzhu Lu

**Affiliations:** 1 State Key Laboratory of Tree Genetics and Breeding, Key Laboratory of Tree Breeding and Cultivation of the State Forestry Administration, Research Institute of Forestry, Chinese Academy of Forestry, Beijing 100091, China; wlj307@caf.ac.cn (L.W.); hujjlys@gmail.com (J.H.); tangfangcaf2@126.com (F.T.); 2 Co-Innovation Center for Sustainable Forestry in Southern China, Nanjing Forestry University, Nanjing 210037, China; 3 The Key Laboratory of Forest Protection, State Forestry Administration, Research Institute of Forest Ecology, Environment and Protection, Chinese Academy of Forestry, Beijing 100091, China; qulj2001@caf.ac.cn; 4 Key Laboratory of Forest Genetics & Biotechnology, Ministry of Education, Nanjing Forestry University, Nanjing 210037, China; zhanglwnl@126.com

**Keywords:** induced resistance, poplar, insects, *Apripona germari*, metabolomics

## Abstract

Plants have developed biochemical responses to adapt to biotic stress. To characterize the resistance mechanisms in poplar tree against *Apripona germari*, comprehensive metabolomic changes of poplar bark and xylem in response to *A. germari* infection were examined by gas chromatography time-of-flight mass spectrometry (GC–TOF/MS). It was found that, four days after feeding (stage I), *A. germari* infection brought about changes in various metabolites, such as phenolics, amino acids and sugars in both bark and xylem. Quinic acid, epicatechin, epigallocatechin and salicin might play a role in resistance response in bark, while coniferyl alcohol, ferulic acid and salicin contribute resistance in xylem. At feeding stages II when the larvae fed for more than one month, fewer defensive metabolites were induced, but levels of many intermediates of glycolysis and the tricarboxylic acid (TCA) cycle were reduced, especially in xylem. These results suggested that the defense strategies against *A. germari* might depend mainly on the early defense responses in poplar. In addition, it was found that bark and xylem in infected trees accumulated higher levels of salicylic acid and 4-aminobutyric acid, respectively, these tissues displaying a direct and systemic reaction against *A. germari*. However, the actual role of the two metabolites in *A. germari*-induced defense in poplar requires further investigation.

## 1. Introduction

The poplar tree (*Populus* spp*.*), a fast-growing species with high timber yields, play important roles in wood production and ecological maintenance, and are widely distributed in China [1]. However, the poplar plantations are frequently threatened by stem borers. Asian longhorned beetle, *Apriona germari* (Hope), is a destructive stem-boring pest in China. The main damage associated with *A. germari* is caused by the larvae, which create long tunnels in the trunk. This affects the growth of the trees, increases the chances of windbreak and decreases the quantity and quality of the timber. Notably, during population outbreaks, the insects are capable of causing extensive mortality and severe damage to poplar plantations. Breeding for resistance is considered as an economical and environment-friendly approach to manage *A. germari* [2]. Knowledge of the mechanisms underlying the insect resistance will facilitate the breeding of resistant cultivars.

It is well known that plants have evolved a variety of strategies for defending themselves against insects. Chemical defense is one of the most effective strategies for insect control because metabolites play important roles in the resistance reaction [3]. Comparing with the constitutive barriers, the induced chemical defenses require lower resource allocation costs, and are considered to be advantageous for plant fitness [4,5]. In poplar trees, studies on induced chemical defense have focused mainly on leaf-chewing insects. For example, larvae of aspen leaf miner (*Phyllocnistis populiella*) induced increased levels of tremulacin and salicortin in aspen leaves [6]. Feeding of forest tent caterpillar (*Malacosoma disstria*) and leaf beetle (*Chrysomela confluens*) larvae significantly increased condensed tannin concentrations in poplar leaves [7]. However, until now, investigations on induced chemical defense to the longhorned beetle in poplar trees have not been reported. Previous studies showed that salicin and salicortin were involved in the constitutive resistance in poplar against the stem borer *Anoplophora glabripennis* [8]. However, whether these metabolites also function as induced defenses has not been investigated.

Metabolomics, a post-genomics tool, can elucidate the host biochemical responses to exogenous elicitors by comprehensively analysis of all metabolites in plant. A systematic analysis on plant metabolism upon insect challenge allows the identification of defensive compounds, revealing novel pathways and discrimination the mechanistic responses to different elicitors [9,10]. In fact, many studies on insect-plant interactions have proved that metabolomics is a well-established and valuable method to determine plant responses to insect feeding and identify the defensive metabolites [11,12,13]. Compared with the traditional targeted chemical analyses which focus on one particular class of chemicals in plant and only measure a tiny fraction of all metabolites, metabolomics has further extended to multiple chemical groups (including unknown metabolites), giving a comprehensive untargeted chemical analysis on the defense responses. Therefore, metabolomic analysis on *A. germari*-induced defense response in the poplar tree will be helpful for exploring underlying defense mechanisms.

In the present study, *A. germari* induced metabolic responses were investigated in poplar clone DH (*P. deltoids* cv. “Danhong”), which have been widely planted in China. Non-targeted metabolic profiling of poplar bark and xylem, with and without *A. germari* infection, were analyzed by gas chromatography time-of-flight mass spectrometry (GC–TOF/MS). The results revealed that upon infecting the tree, the insect induced metabolic changes in the tree to satisfy nutritional requirements; conversely, there was a shift in the tree’s metabolism to defend against attack. The levels of phenolics, amino acids and sugars were significantly disturbed by *A. germari* in both bark and xylem. The defense strategies against *A. germari* might depend on early defense responses in poplar because more defensive metabolites were induced at earlier feeding stages. Quinic acid, epicatechin, epigallocatechin and salicin might play a role in resistance response in bark, while coniferyl alcohol, ferulic acid and salicin contributed to resistance in the xylem. The results will be helpful for identifying metabolic biomarkers for inductive resistance to the insect and may help us to gain an improved understanding of the molecular mechanisms underlying resistance of trees to *A. germari*.

## 2. Results

### 2.1. Metabolic Profiles of Bark and Xylem Tissues in A. germari and Mock Infected Trees

**Figure 1:**
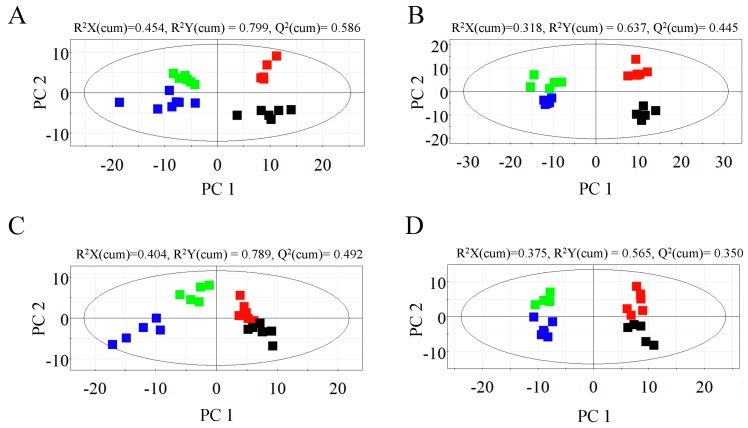
PLS-DA score plots derived from spectra of mock- and *A. germari*-infected samples. (**A**,**B**) Score plots for the polar (**A**) and lipophilic profiles (**B**) for bark samples; (**C**,**D**) Score plots for the polar (**C**) and lipophilic profiles (**D**) for xylem samples. R^2^X (cumulative) and R^2^Y (cumulative) represented the cumulative fraction of the x and y variance in the model, respectively. Q^2^ (cumulative) reflects the predictive capacity of the model. Black and red dots represented mock- and *A. germari*-infected samples, respectively, at stage I. The green and blue squares represented mock- and *A. germari*-infected samples, respectively, at stage II.

To investigate the *A. germari* induced metabolic responses in poplar, metabolic profiles of bark and xylem tissues with and without infection, were analyzed by GC–TOF/MS. In bark tissues, 1055 peaks were obtained, in which 412 and 643 peaks were identified in the polar and lipophilic phases, respectively. In xylem tissues, 784 peaks were measured, with 305 peaks being identified in the polar phase and 305 peaks in the lipophilic phase. A library search using the NIST mass spectral data base showed that the metabolites in the lipophilic phases included phenolic acids, benzenoids, sterols, fatty acids and their derivatives, while those in polar phases mainly included amino acids, sugars, organic acids and their derivatives. Metabolic profiles of the mock- and *A. germari*-infected samples were differentiated by PLS-DA model in SIMCA-P 11.5 (Umetrics, Umeå, Sweden). As shown in Figure 1, the first principal component PC 1 was dominated by the developmental stages, while the second principal component PC 2 was dominated by *A. germari* infection. At feeding stage I, four days after the larva feeding, the infected bark tissues were clearly separated from their mock controls, but the two groups of the xylem tissues were only just distinguishable, indicating that more metabolic changes occurred in the bark than the xylem. At feeding stage II when the larvae fed for more than one month, separations were observed between the infected bark and the xylem tissues with their corresponding controls (Figure 1), but separations in the case of the polar metabolite profiles for the infected xylem and controls were more clearly discernible.

### 2.2. Metabolic Alteration in Bark and Xylem Tissues Following Infection of A. germari

The infected materials at each sampling stage were compared with a mock control based on the loadings plot from the PLS-DA model. Statistically significant metabolites that helped distinguish between the infected and mock groups were obtained according to two criteria, namely Mann–Whitney *p*-value <0.05 and variable importance value >1.0. Comparing the spectra of the metabolites with those from the commercial NIST library, the differential metabolites were tentatively identified.

**Figure 2:**
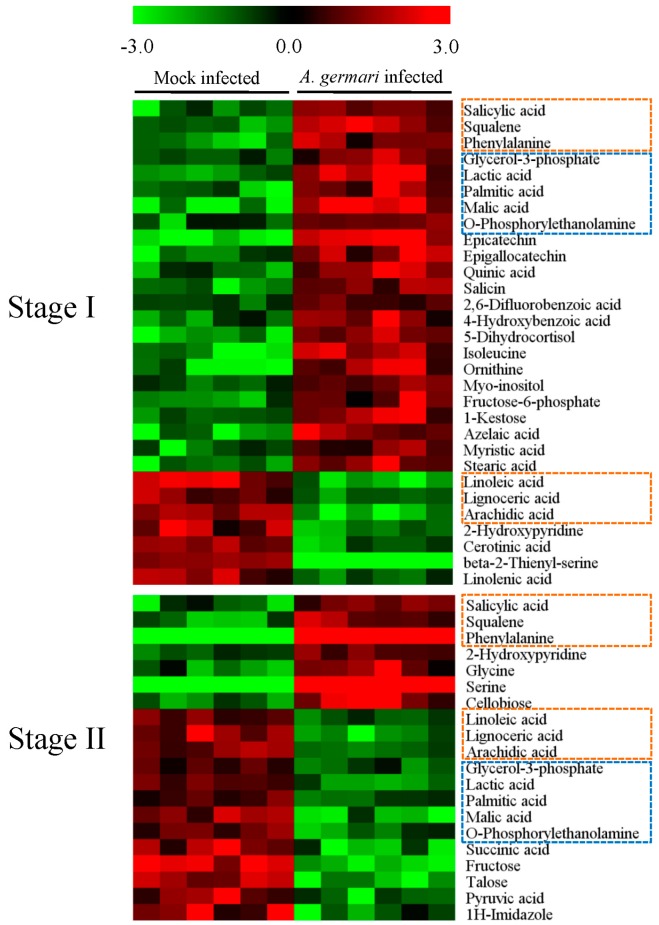
Differential metabolites associated with *A. germari* infection in bark tissues. Black bars denoted the sample classes. The columns and rows represented the individual tissue samples and distinct metabolites, respectively. The elevated and decreased levels of a metabolite were showed by increases in the intensities of red and green, respectively. Metabolites which appeared in both stages were marked with boxes. The orange and blue boxes marked metabolites showed similar and opposite changes between stage I and II, respectively.

**Figure 3:**
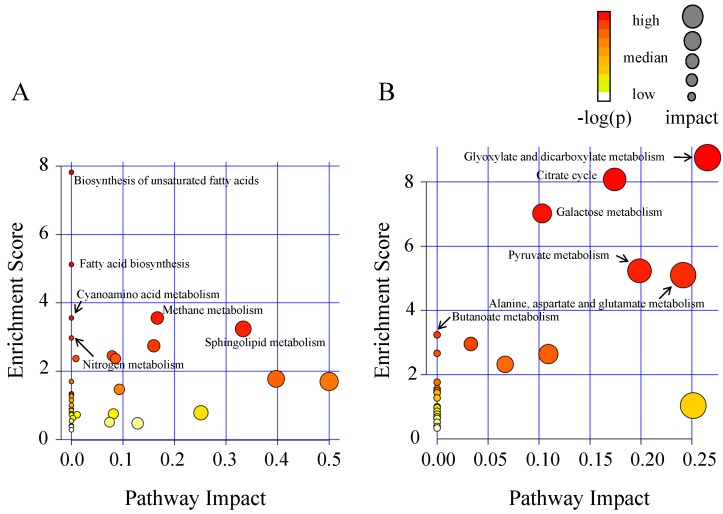
Schematic of the metabolome based on the metabolic pathways mapped with the *A. germari* infection associated differential metabolites. The analysis was performed using MetaboAnalyst software. (**A**) Pathway assignment of differential metabolites in bark tissues; (**B**) pathway assignment of differential metabolites in xylem tisseus.

In bark tissues, 26 differential metabolites were identified when comparing mock-infected samples between stage I and II, of which 15 metabolites were disturbed by *A. germari* infection (Figure S1A). Comparation of the *A. germari*-infected samples with their mock controls revealed that, 30 metabolites were found to be disturbed by the presence of *A. germari* at feeding stage I. Twenty-three metabolites were up-regulated including phenolics, fatty acids, sugars, amino acids, sterols, their substrates and derivatives. Seven metabolites (linoleic acid, arachidic acid, linolenic acid, β-2-thienyl-serine, lignoceric acid, cerotinic acid and 2-hydroxypyridine) were down-regulated (Figure 2). However, at feeding stage II, the number of the altered metabolites was less. Among the 20 disturbed metabolites, 7 metabolites were increased while 13 metabolites were decreased. Six metabolites (phenylalanine, salicylic acid, squalene, arachidic acid, linoleic acid and lignoceric acid) that were up- or down-regulated at stage I showed similar changes at stage II. Five metabolites (lactic acid, malic acid, glycerol-3-phosphate, palmitic acid and *O*-phosphorylethanolamine) were increased at 20 days but decreased at 60 days. Pathway enrichment and topological analyses revealed that *A. germari* induced metabolites in bark were mainly assigned to biosynthetic pathways for unsaturated fatty acids, fatty acid biosynthesis, cyanoamino acid metabolism, methane metabolism, sphingolipid metabolism and nitrogen metabolism (Figure 3).

**Figure 4:**
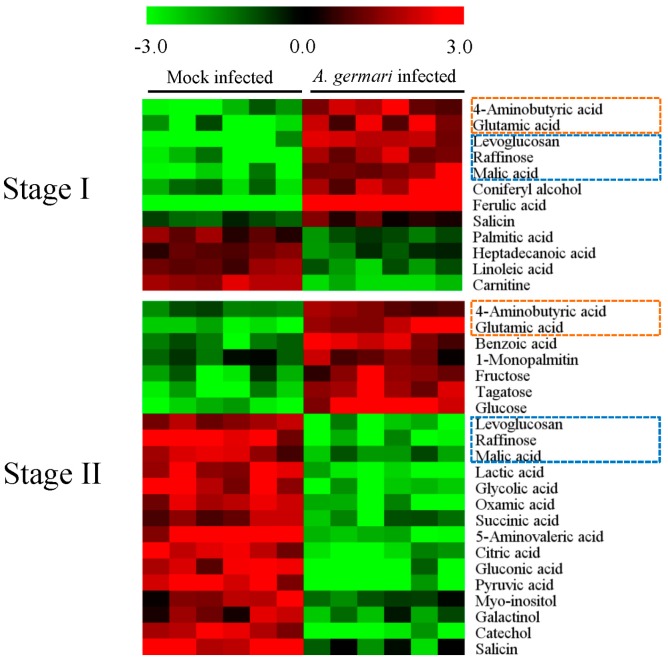
*A. germari* induced differential metabolites in xylem tissues. Black bars denoted the sample classes. The columns and rows represented the individual tissue samples and distinct metabolites, respectively. The elevated and decreased levels of a metabolite were showed by increases in the intensities of red and green, respectively. Metabolites which appeared in both stages were marked with boxes. The orange and blue boxes marked metabolites showed similar and opposite changes between stage I and II, respectively.

In xylem tissues, there are 27 identified differential metabolites between mock-infected samples at stage I and II. In these differential metabolites, 15 metabolites were disturbed by *A. germari* infection at stage I or II (Figure S1B). At feeding stage I, 12 metabolites were disturbed. The metabolites that were increased in concentration included phenolics (coniferyl alcohol, ferulic acid and salicin), sugars (levoglucosan and raffinose) and amino acids (glutamic acid and 4-aminobutyric acid) and an intermediate in the citric acid cycle (malic acid). In contrast, the metabolites that decreased in concentration included fatty acids (palmitic acid, heptadecanoic acid and linoleic acid) and a metabolite associated with fatty acid metabolism (carnitine) (Figure 4). More than 1 month later, at feeding stage II, more metabolomic variations were observed in the xylem. Among these variations, the number of metabolites that had decreased substantially outnumbered those that had increased. Fifteen metabolites, including two phenoloics (catechol and salicin), nine organic acids (lactic acid, oxamic acid, succinic acid, glycolic acid, malic acid, 5-aminovaleric acid, gluconic acid, citric acid and pyruvic acid) and four carbohydrates (levoglucosan, myo-inositol, galactinol and raffinose) were significantly decreased. However, only seven metabolites (fructose, tagatose, glucose, 1-monopalmitin, 4-aminobutyric acid, glutamic acid and benzoic acid) were significantly induced. These metabolites were mainly enriched in the pathways of the citrate cycle (TCA cycle), pyruvate metabolism, glyoxylate and dicarboxylate metabolism, butanoate metabolism, alanine, aspartate and glutamate metabolism and galactose metabolism (Figure 3).

## 3. Discussion

In this study, the metabolic responses of poplar to *A. germari* at the early feeding stage (stage I) and later stage (stage II) were investigated using comprehensive untargeted chemical analysis. Metabolic profiles of bark and xylem near the infection sites were analyzed, respectively, giving more detailed information on metabolic changes triggered by *A. germari* in poplar.

Before ovipositing, the female adult bit through the bark and gnawed a nidus within which an egg was laid in the xylem. Therefore, early metabolic changes in infected trees might be associated with the damage caused by the adult and feeding of larvae. At stage I in this study, metabolic analysis of samples revealed that primary and secondary metabolisms in the tree were significantly disturbed by *A. germari*. Especially, the amounts of some sugars, amino acids and fatty acids were significantly changed both in the bark and the xylem. Sugars and amino acids are important nutritional components assimilated by the insect from the plant, while amino acids and fatty acids are precursors of some defensive metabolites. The alteration in the metabolism of these metabolites might be associated with a reallocation of nutrients and provision of precursors for the biosynthesis of defensive metabolites and indicate that the insect might induce some egocentric changes in the host to satisfy nutritional requirements, and conversely, the tree may switch the metabolism to synthesize defensive metabolites, such as tannins in bark and phenolics involved in lignin synthesis in xylem, thus attempting to defend itself from the insect [3]. Salicin is a key component of constitutive resistance in trees against stem-boring insects [8]. The present study found that the levels of salicin in bark and xylem were clearly increased as a result of infection by *A. germari* indicating that this metabolite might be also involved in the induced resistance to stem borers. In contrast to salicin, some secondary metabolites were induced by *A. germari* in specific tissue possibly resulting from different chemical components in bark and xylem. For example, quinic acid, epicatechin and epigallocatechin were increased in infected bark, while ferulic acid and coniferyl alcohol were up-regulated in infected xylem. Quinic acid, epicatechin, epigallocatechin are low molecular weight tannins and have been shown to be toxic to several herbivores [14,15]. Ferulic acid and coniferyl alcohol are intermediates of the lignification process in wood development. The latter is derived from the former and is monomers of G units in lignin [16]. Lignin and other phenolics derived from lignin synthesis can strengthen plant cell walls. The strengthened cell walls is difficult to digest and, therefore, can be anti-nutritional for insects [17,18]. Furthermore, increased lignin deposition might produce negative effects to insect fitness because phenoloxidase enzymes and toxic by-products (reactive oxygen species and peroxides) are also generated in the process of lignin polymerization [19,20]. Therefore, *A. germari* induced accumulation of the two group metabolites might be important resistance factors of poplar clone DH against this insect in bark and xylem, respectively.

**Figure 5:**
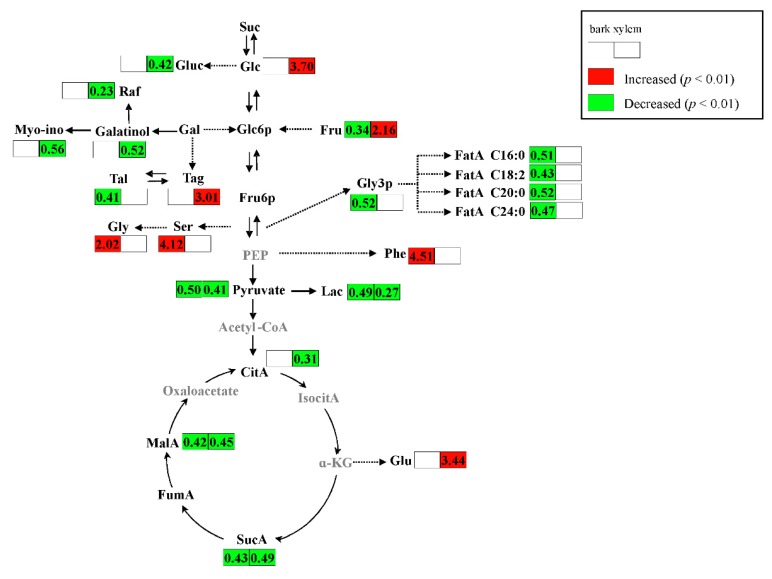
Level changes of metabolites in the main metabolic pathways. Values in the boxes were ratios of peak intensity of metabolites in *A. germari* infected samples to those in mock-infected samples. Colors of red and green represented elevated and decreased levels of a metabolite in *A. germari* infected samples compared with mock-infected samples, respectively. Extended metabolites were marked with gray color. α-KG, alpha-ketoglutarate; CitA, citrate; FatA C16:0, palmitic acid; FatA C18:2, linoleic acid; FatA C20:0, arachidic acid; FatA C24:0, lignoceric acid; Fru, fructose; Fru6P, fructose 6-phosphate; FumA, fumaric acid; Gal, galactose; Glc, glucose; Gluc, Gluconic acid; Glc6P, glucose-6-phosphate; Glu, glutamate; Gly, glycine; Gly3P, glycerol-3-phosphate; IsocitA, isocitric acid; Lac, lactic acid; MalA, malic acid; Myo-ino, Myo-inositol; PEP, phosphoenol-pyruvate; Phe, phenylalanine; Raf, raffinose; Ser, serine; Suc, sucrose; SucA, succinate; Tag, Tagatose; Tal, Talose. Solid and dashed arrows indicated single and multiple enzymatic conversions, respectively.

After feeding for more than one month, the larvae of *A. germari* fed in the pith. The metabolic changes in bark were decreased while those in xylem were increased. Less defensive metabolites were induced by the infection both in bark and xylem. The metabolites that were perturbed were mainly sugars, amino acids, organic acids and fatty acids which were associated with primary metabolism in the tree. As shown in Figure 5, most of the perturbed metabolites in the main metabolic pathways were down-regulated. Especially, several intermediates (pyruvic acid, malic acid, citric acid and succinic acid) in glycolysis and the TCA cycle were clearly decreased in the infected trees indicating primary metabolisms in the tree were weakened at feeding stage II. Glycolysis and TCA cycle, two important central metabolic pathways, provide energy and precursors for the synthesis of primary and secondary metabolites, and are involved in a wide range of physiological functions [21,22,23]. Down-regulation of glycolysis and the TCA cycle implies a disruption of energy production and an extensive disturbance in normal metabolism of the tree, suggesting that the defense system failed and the physiology of the tree was severely affected when the larvae fed in the pith.

Pathway enrichment and topology analyses revealed that the perturbed metabolites enriched pathways in bark tissues were not the same as those in xylem indicating that *A. germari* induced metabolic responses in the two tissues were somewhat different. Specifically, some tissue-specific metabolic changes were found in the present study. For example, in the bark, salicylic acid (SA) was significantly up-regulated by *A. germari* infection at all sampling stages. Meanwhile, the amount of linolenic acid, the precursor of jasmonic acid (JA), decreased in the infected bark although several fatty acids had increased. SA and JA usually act as signaling molecules in the regulation of induced responses to biotic stress. Generally, the former regulates the responses to pathogens, while the latter plays a role in responses to chewing herbivores [24,25,26,27]. However, some recent studies actually found that SA were involved in resistance to phloem feeding aphids [28,29]. Additionally, earlier studies showed that SA accumulated in response to oviposition by insects, both in local and systemic tissues, and activated a response similar to systemic acquired resistance (SAR). The egg-induced SAR defended the feeding larvae by reducing detrimental effects of bacterial pathogens [30]. Indeed, two metabolites (azelaic acid and glycerol-3-phosphate) might undertake this role as SAR mobile signals were also increased in the infected bark at stage I as compared with the control. Therefore, the actual role of salicylic acid in *A. germari* induced responses in poplar has to be corroborated by further studies. In the infected xylem at feeding stages I and II, the contents of glutamic acid and 4-aminobutyric acid (GABA), which is a non-protein amino acid derived from glutamic acid, were all significantly induced by *A. germari*. GABA accumulation can be stimulated by feeding of leaf eating larvae and thus affect larval performance possibly as the ingested GABA interferes with the normal development of the insects [31,32,33,34]. Additionally, the accumulation of GABA is independent on herbivore defense related phytohormones, jasmonates [35]. Thus, *A. germari* induced accumulation of GABA in xylem suggested that GABA might also be involved in plant defense against stem-boring insects.

## 4. Materials and Methods

### 4.1. Insect Bioassays

The poplar clone DH (*P. deltoids* cv. “Danhong”), which is widely planted in China, was chosen to investigate the *A. germari* induced metabolic changes in poplar. Tissues of DH were obtained by vegetative propagation using cuttings which were grown in a greenhouse (23 ± 1 °C; light:dark = 16:8 h). When the height reached 20–35 cm, the poplars were transferred to an open-air garden. We selected for bioassay and metabolic profiling 16 two-year-old trees (1.3 m above the ground: diameter 4.5 ± 0.3 cm; height about 4 m) that lacked any signs or symptoms of pathogen or insect attack (eight corresponding to *A. germari* infected and eight mock infected). In early July, the female and male *A. germari* adults were captured from paper mulberry (*Broussonetia papyrifera*) and were placed in wire cages (diameter 20 cm; length 60 cm) which were fixed in the trees at 2 m above the ground. One male and 1 female were placed in each cage. Fresh branches of paper mulberry tree were supplied to the adults and were replaced every 2 days. The mock-infected trees received fixed wire cages but without insects.

Approximately 6 days after mating with the male, the female gnawed the nidus and laid an egg in the xylem. When three nidi were produced in one tree, the adults were removed to permit an appropriate induction by the larvae in the tree. Ten days later, 90% of the larvae hatched and fed first in the outer xylem tissue of the tree. Fresh frass of the larvae was first observed after feeding for 4 days. Then, bark (including phloem and cambium) and 3-mm-thick outer xylem tissues near the dejection site under which the larvae hid in the infected trees and those in the corresponding altitude of the mock infected trees were sampled. Hereafter, this sampling stage is referred to as stage I. Forty days later, the larvae bored into the pith of the tree, with small frass expulsion holes made through the bark. Tissues of infected bark and xylem and their corresponding mock controls were also sampled. The second sampling stage is referred to as stage II. After removal from the tree, the bark and the xylem were separated and then frozen immediately in liquid nitrogen.

### 4.2. Metabolite Extraction and Derivatization

Metabolites in bark and xylem tissues were extracted according to the protocol of Weckwerth *et al.* and Qu *et al.* with some modifications [36,37]. The frozen tissues were ground to powder using a mortar and pestle. Approximately 100 mg of the powder was mixed with 1.5 mL methanol:chloroform:water (5:2:2). The internal quantitation standards, nonadecanoic acid (5 μL of 2.0 mg/mL) and ribitol (80 μL of 0.2 mg/mL) were added to the mixture. Then, the mixture was placed in a supersonic extractor and extracted for 40 min. The suspension was centrifuged (10,000× *g*, 10 min) and 1.0 mL supernatant was collected. Five hundred microliters distilled water and 400 μL chloroform were mixed with the supernatant and then centrifuged (5000× *g*, 5 min). Finally, five hundred microlitres polar extracts and 200 μL lipophilic extracts were taken out and dried using a nitrogen gas stream in a vacuum rotary evaporator without heating. Fifty microliters of methoxyamine hydrochloride (Sigma-Aldrich, St. Louis, MO, USA, 20 mg/mL) dissolved in pyridine (ACS grade) were added to the dried extracts and incubated for 2 h (30 °C). Then, 100 μL of MSTFA (*N*-Methyl-*N*-trifluoroacetamide) was mixed with the above solution and incubated for 30 min (37 °C).

### 4.3. Analysis of Metabolites by GC–TOF/MS

The derivatized samples were analyzed using a LECO Pegasus IV GC-TOF/MS system. An Agilent 6890 gas chromatograph (Agilent, San Jose, CA, USA) was used with a 30 m long, 0.25 mm i.d. (inside diameter) fused silica capillary column with 0.25 μm DB-5 MS stationary phase (J & W Scientific, Folsom, CA, USA). One μL was injected in splitless mode and 10:1 split ratio for lipophilic and polar extracts, respectively. Chromatography was performed at a flow rate of 1.5 mL/min. For the lipophilic metabolites, the initial column temperature was set as 100 °C and maintained for 2 min, 100 to 200 °C at 8 °C/min and held for 1 min, 200 to 260 °C at 3 °C/min and held for 1 min, and then 260 to 320 °C at 8 °C/min and held for 4 min. For the polar metabolites, the initial column temperature was set as 100 °C and maintained for 2 min, 100 to 260 °C at 4 °C/min and held for 1 min, 260 to 320 °C at 20 °C/min and held for 3 min. The column’s effluent was then directed to the ion source of a Leco Pegasus IV time off light mass (TOF) spectrometer with an ion source temperature of 200 °C, transferline temperature of 250 °C, and electron ionization at −70 eV. Mass spectra were obtained from *m*/*z* 80–500 at 20 scans per second. The detector voltage was set to 1800 V.

### 4.4. Data Processing and Statistical Analyses

ChromaTOF version 4.0 (LECO, St. Joseph, MI, USA) was used for the raw data processing. Weights of the samples were normalized to the maximum value to minimize the discrepancy caused by sample weights. Then, the data was normalized using min-max normalization. After normalization, the missing values for each metabolite were replaced with the observed minimum value. Multivariate and univariate statistical techniques were used to analyze the data. The multivariate analyses were performed by the software package SIMCA-P 12.0 (Umetrics, Umeå, Sweden). Using the autofit function, PLS-DA model was fitted on mean centered unit variance scaled data. Potential differential metabolites were extracted according to the variable importance plot (VIP) values (VIP > 1). Meanwhile, the non-parametric Mann–Whitney test (SPSS17.0, IBM Corporation, Chicago, IL, USA) was used to distinguish differential metabolites (*p* < 0.01). Compounds were tentatively identified using NIST standard mass spectral databases (similarity ≥ 80%). Heat maps derived from the log-transformed normalized data were obtained using the cluster program and visualized using the Mev software v4.9 [38]. A red/green color scheme in the heat maps represented increase and decrease of level of metabolite relative to the median concentration, respectively. Pathway enrichment analysis of the metabolites was performed using MetaboAnalyst 3.0 [39]. The out-degree centrality algorithms and hypergeometric test were employed based on the *Arabidopsis thaliana* pathway library.

## 5. Conclusions

This study traced the metabolic responses in bark and xylem of poplar trees against infection by *A. germari* at earlier and later stages (stages I and II) of feeding. It was observed that the primary and secondary metabolisms in bark and xylem tissues in infected trees were significantly altered by *A. germari.* More defensive metabolites were induced at the earlier feeding stage (stage I) than the later stage (stage II) when the energy production of the tree was disrupted. The defensive compounds induced in bark were quinic acid, epicatechin, epigallocatechin and salicin, while those in xylem include coniferyl alcohol, ferulic acid, salicin and GABA. The present results may prove to be useful in identifying the metabolic biomarkers for *A. germari* resistance in poplar and to gain a better understanding of the mechanistic basis for resistance against stem-boring pest in trees.

## Supplementary Materials

Supplementary materials can be found at http://www.mdpi.com/1422-0067/17/6/923/s1.
