# Supplementary material for: Metabolic Responses of Poplar to Apripona germari (Hope) as Revealed by Metabolite Profiling
Source: Int J Mol Sci. 2016 Jun 20;17(6):923. doi: 10.3390/ijms17060923 (PMC4926456; doi:10.3390/ijms17060923)
Supplement: Supplementary file 1 [file ijms-17-00923-s001.pdf]

Supplementary Materials: Metabolic Responses of Poplar to *Apripona germari* (Hope) as Revealed by Metabolite Profiling

Lijuan Wang, Liangjian Qu, Liwei Zhang, Jianjun Hu, Fang Tang and Mengzhu Lu

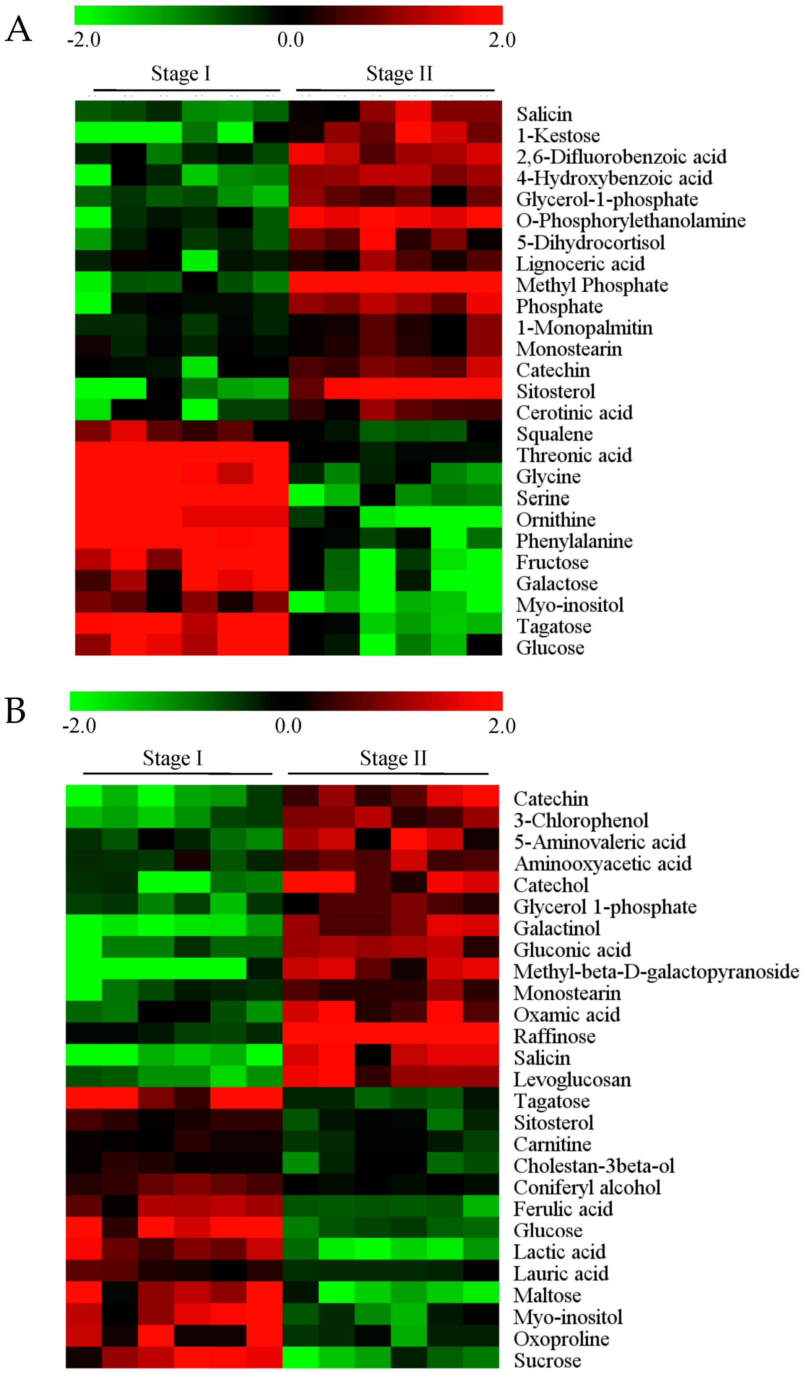

Figure S1. Differential metabolites in mock-infected tissues between feeding stage I and II. (**A**) Differential metabolites detected in mock-infected bark tissues; (**B**) differential metabolites detected in mock-infected xylem tissues. Black bars denoted the sample classes. The columns and rows represented the individual tissue samples and distinct metabolites, respectively. The elevated and decreased levels of a metabolite were showed by increases in the intensities of red and green, respectively.
